# Graph neural network for integrated water network partitioning and dynamic district metered areas

**DOI:** 10.1038/s41598-022-24201-w

**Published:** 2022-11-14

**Authors:** Minglei Fu, Kezhen Rong, Yangyang Huang, Ming Zhang, Lejing Zheng, Jianfeng Zheng, Mayadah W. Falah, Zaher Mundher Yaseen

**Affiliations:** 1 College of Information Engineering, Zhejiang University of Technology, Hangzhou, 310023 China; 2 Hangzhou Laison Technology Co., Ltd, Hangzhou, 310063 China; 3 Department of Building and Construction Technologies Engineering, AL-Mustaqbal University College, Hillah, 51001 Iraq; 4 New Era and Development in Civil Engineering Research Group, Scientific Research Center, Al-Ayen University, Thi-Qar, 64001 Iraq

**Keywords:** Civil engineering, Applied mathematics, Computational science

## Abstract

Water distribution systems (WDSs) are used to transmit and distribute water resources in cities. Water distribution networks (WDNs) are partitioned into district metered areas (DMAs) by water network partitioning (WNP), which can be used for leak control, pollution monitoring, and pressure optimization in WDS management. In order to overcome the limitations of optimal search range and the decrease of recovery ability caused by two-step WNP and fixed DMAs in previous studies, this study developed a new method combining a graph neural network to realize integrated WNP and dynamic DMAs to optimize WDS management and respond to emergencies. The proposed method was tested in a practical case study; the results showed that good hydraulic performance of the WDN was maintained and that dynamic DMAs demonstrated excellent stability in emergency situations, which proves the effectiveness of the method in WNP.

## Introduction

A water distribution network (WDN) is composed of demand nodes and supply pipelines and provides water to domestic and nondomestic users. WDN is managed by water distribution companies. Owing to pipe bursts, connection leakages, water theft, and other factors, WDNs are susceptible to significant unexpected water consumption^1^. The British Water Industry Association proposed the concept of water network partitioning (WNP), which divides a WDN into several district metered areas (DMAs) to more effectively manage the WDS^2^, an approach which has proven to be of great benefit to the control of water leakage^3–8^.

Dividing a WDN into several DMAs has many benefits, including but are not limited to reducing leakage, reducing the flow of contaminants^9–14^, optimising pressure management^15–17^ and helping to repair the water pipe network in emergencies^18^. However, WNP has some drawbacks, primarily the associated economic cost, deterioration of water quality^19,20^ and reduced capacity to respond to abnormal situations^21,22^. Further, research has shown that the disadvantages of WNP can be overcome by using methods such as dynamic DMAs management and other technologies^21^.

Normally, WNP is accomplished in two phases: clustering and dividing. The methods and concepts applied in the clustering phase include graph theory^23–31^, community structure^32–34^, modularity-based algorithms^35^, multi-level partitioning^36,37^, spectral graph algorithms^38–40^ and multi-agent approaches^41,42^. The methods applied in the dividing phase include single-objective programming^10,17,27,43^, multiple-objective programming^44–48^, iterative methods^17,32^ and heuristic algorithms^49^. The above methods can perform WNP, simplify management, monitor sudden leakage, and control the flow rate of contaminants, but the following two limitations need to be addressed:

- Using two phases and different sets of objectives reduces the search range of the global optimal solution.
- The use of a fixed boundary causes a water pressure drop owing to the reduction of inflow in the emergency.

Integrated WNP and dynamic DMAs are solutions to the above two problems. These approaches are used for the integrated establishment of DMAs and dynamic management of DMA boundaries, to achieve intelligent and flexible WDS management. In this study, an integrated WNP and dynamic DMAs method were developed, and simulating these methods in a network and proving the advantages of the method, which can maintain the normal operation of a network in an emergency. The contributions of this study are as follows:

- Graph neural network used for the first time in water network partitioning.
- Dynamic district metered areas are used to optimise water network management.
- A simulation shows that this method is efficient and highly resilient to emergencies.

## Related works

**Figure 1:**
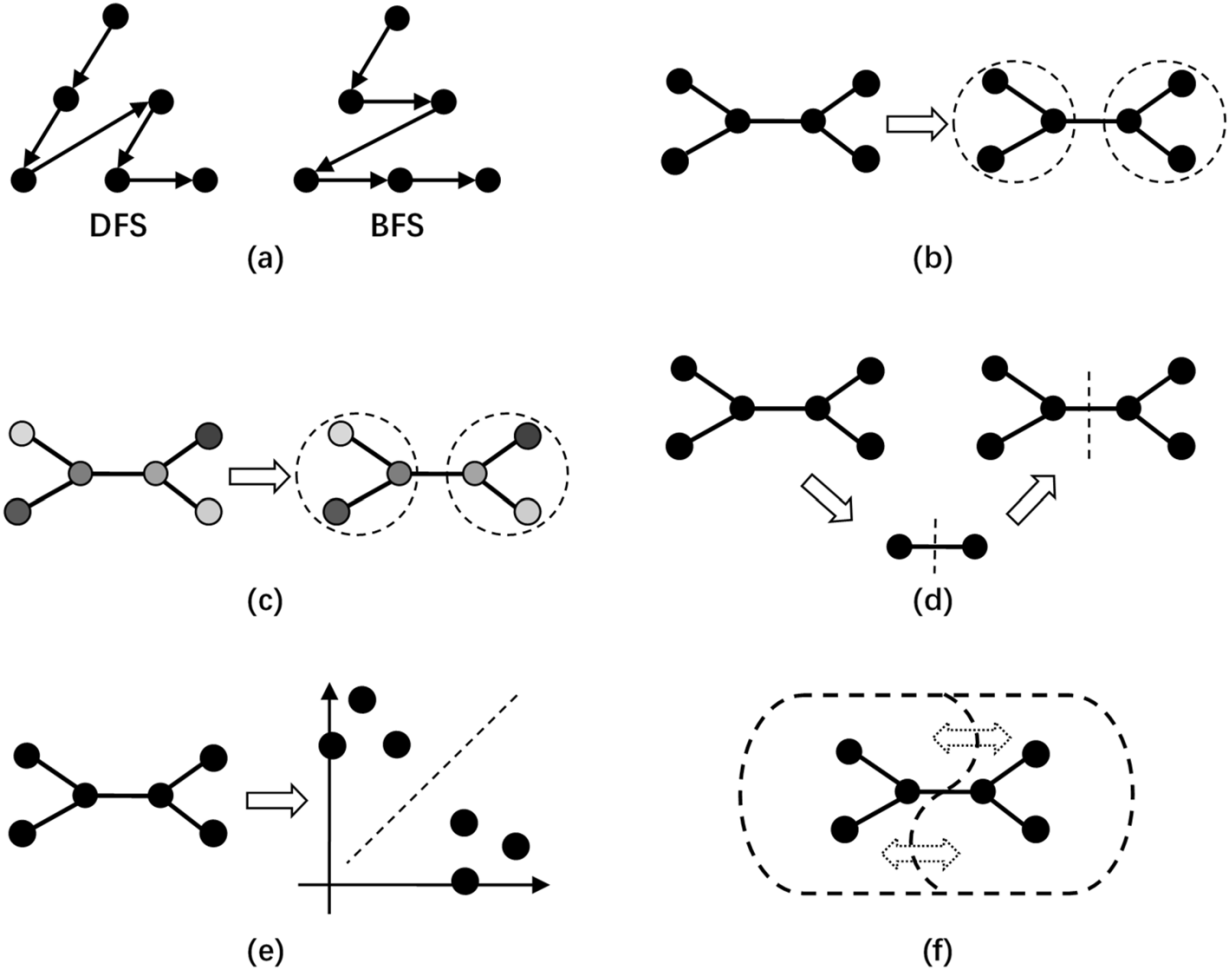
Traditional water network partitioning methods: (**a**) graph theory methods, (**b**) community structure algorithm, (**c**) modularity-based algorithm, (**d**) multi-level partition algorithm, (**e**) spectral graph algorithm, and (**f**) multi-agent approach.

Previous studies have focused on clustering and dividing algorithms. The former refers to clustering WDN into DMAs based on the attributes of demand nodes and pipeline connections, The International Water Association has proposed the implementation of WNP based on administrative boundaries, road conditions, and number of residents^2^. This method is quite straightforward, but it is difficult to apply to a large WDN. In addition, a WNP implemented by the trial-and-error method is usually unreasonable and negatively affects water quality. Tzatchkov et al. introduced graph theory into WNP and proposed a WNP method based on depth-first search (DFS) and breadth-first search (BFS) (Fig. 1a)^24^, which optimized the hydraulic performance of the formed DMAs. Giustolisi and Di Nardo implemented genetic algorithms to accelerate the WNP^20,25^. Perelman et al. proposed using DFS to identify tightly connected pipes and using reverse BFS to identify sparsely connected pipes to establish more reasonable DMA boundaries^26^. Morrison et al. proposed the separation of the main network of the WDN from the branch network^50^, and Campbell combined this idea with graph theory to achieve more reasonable WNP^29,30^.

The core of the above methods is to use graph theory to realize WNP, but WDN is complicated in real-world situations, and it is usually difficult to achieve good results using looped WDN. Diao et al. proposed the use of a community structure algorithm to cluster demand nodes with similar locations (Fig. 1b)^32^, and finally formed DMAs with similar spatial locations. Giustolisi et al. found that the demand nodes’ altitude and consumption will also affect WNP. It is unreasonable to implement WNP based solely on spatial locations. Thus, they proposed implementing a community structure algorithm and proposed a modularity-based algorithm considering the hydraulic performance in WNP (Fig. 1c)^35^. In fact, many methods are available for implementing WNP. Diao et al. proposed a multilevel partition algorithm for implementing WNP (Fig. 1d)^32^. Herrera et al. proposed a WNP method based on a spectral graph algorithm (Fig. 1e)^38^. Izquierdo et al. used multiple interacting agents to cooperate and compete to achieve WNP and proposed a WNP method based on a multi-agent approach (Fig. 1f)^41^.

There are one or more pipe connections between any adjacent DMAs, called boundary pipes. By choosing to install flow meters or valves at the boundary pipes to facilitate subsequent water conservancy monitoring and contaminant control, this process is called the dividing phase. Previous studies proposed some indicators to measure whether the results of dividing are reasonable^51–55^. They also recommended the following steps:

- Maintain the emergency recovery capability of the WDN.
- Maintain the water age in the WDN at an appropriate level.
- Improve uniformity between DMAs.
- Maintain the water pressure of the WDN.

The first goal helps the WDN remain stable under abnormal conditions, the second goal helps maintain the chlorine content in the WDN at an appropriate level, the third goal is conducive to the daily operation and management of WNP, and the fourth goal can ensure that the WNP does not affect residents' daily water use. By optimising one or more targets and using a heuristic algorithm to speed up the optimisation process, the position of the flow meters in the boundary can be determined^45–47^.

In recent years, some research has provided possibilities for new WNP methods. Inspired by word2vec “*word to vector*” model^56^, Perozzi et al. proposed a deep walk^57^, which opened the door to the era of deep learning of using graph neural networks (GNNs). Subsequently, Kip et al. proposed the use of graph convolutional networks (GCN)^58^ and Velickovic et al. proposed the use of graph attention networks (GAT)^59^, which greatly improved the effectiveness of GNN use. At present, GNNs have been effectively used in the fields of recommendation systems, financial risk control, molecular chemistry, traffic prediction, etc.^60–63^. They can also be used for the feature extraction, aggregation, and node classification of WNP. The process of using a GNN for WNP and dynamic boundary management is mainly composed of three logical steps: aggregate node information, integrated WNP, and dynamic boundary management.

## Integrated WNP and dynamic DMAs

**Figure 2:**
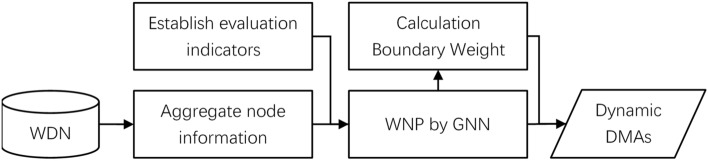
Flowchart of the process for integrated WNP and dynamic DMAs.

This section discusses the feasibility of integrated WNP and dynamic DMAs using a GNN. A WDN can be integrally partitioned through this method and can realize dynamic boundary management to cope with emergencies. The steps of this method for the WDN are shown in Fig. 2.

### Aggregate node information

The WDN can be regarded as a graph $G$ that is composed of ${N}_{n}$ demand nodes and ${N}_{p}$ pipes. The WNP needs to classify demand nodes with the same attributes as a DMA. These attributes include the longitude, latitude, and altitude of the demand nodes. At the same time, it is necessary to maintain the balance of water consumption and water pressure of each DMA, which is conducive to the daily monitoring and maintenance of the WDS.

The process of aggregate node information involves adding the attributes of the node itself and its neighbouring nodes, then using the sum of the addition as the attribute of the node in the next graph ${G}_{1}$, and finally forming the new node attribute graph ${G}_{1},{G}_{2}\dots {G}_{k}$.

**Figure 3:**
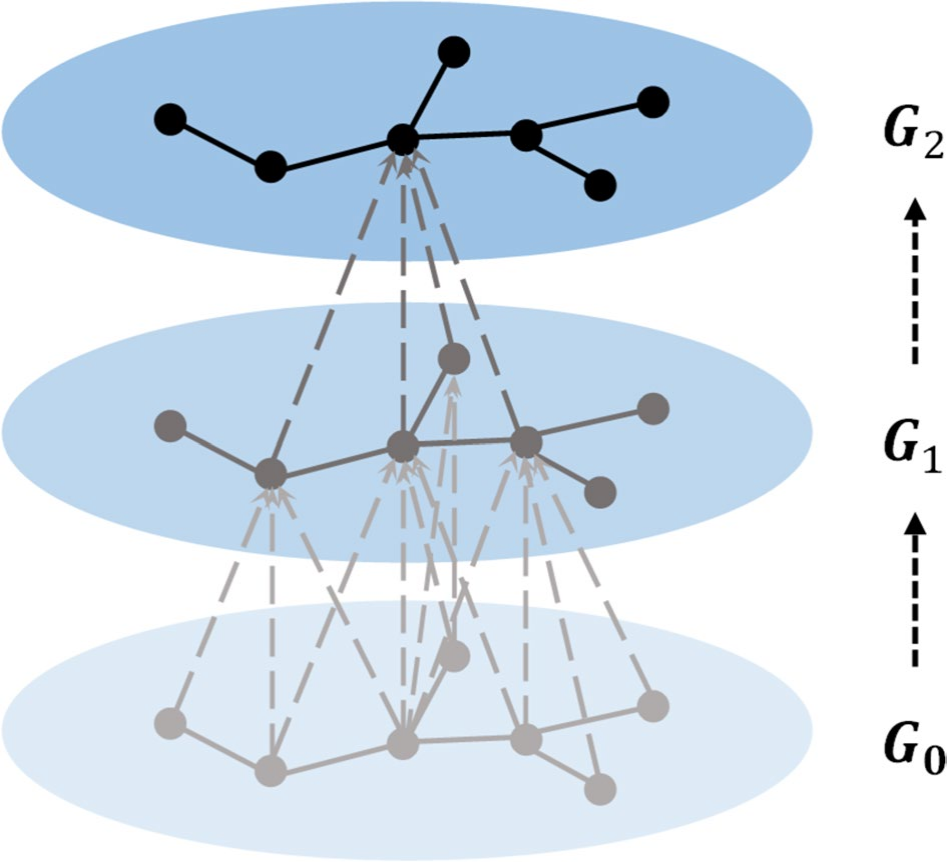
Schematic of aggregate node information when $k=2$.

When using the process of $k$ iterations of aggregation, each demand node contains the attributes of its neighbouring nodes of order $k$. If $k$ is too small, the range of aggregation will shrink excessively, and consequently, the neighbouring information cannot be extracted effectively. If $k$ is too large, the attributes of nodes tend to be more similar, and consequently, these nodes cannot be perfectly distinguished. According to related research^64^, $k$ is usually 2, 3 or 4. The aggregation process at $k$ = 2 is illustrated in Fig. 3.

### Integrated WNP using GNN

According to the results obtained from aggregate node information in Sect.  3.1, the following steps must be performed to classify nodes by using unsupervised learning and forming several DMAs.

- Build neural networks
- Establish the evaluation index
- Train neural networks

**Figure 4:**
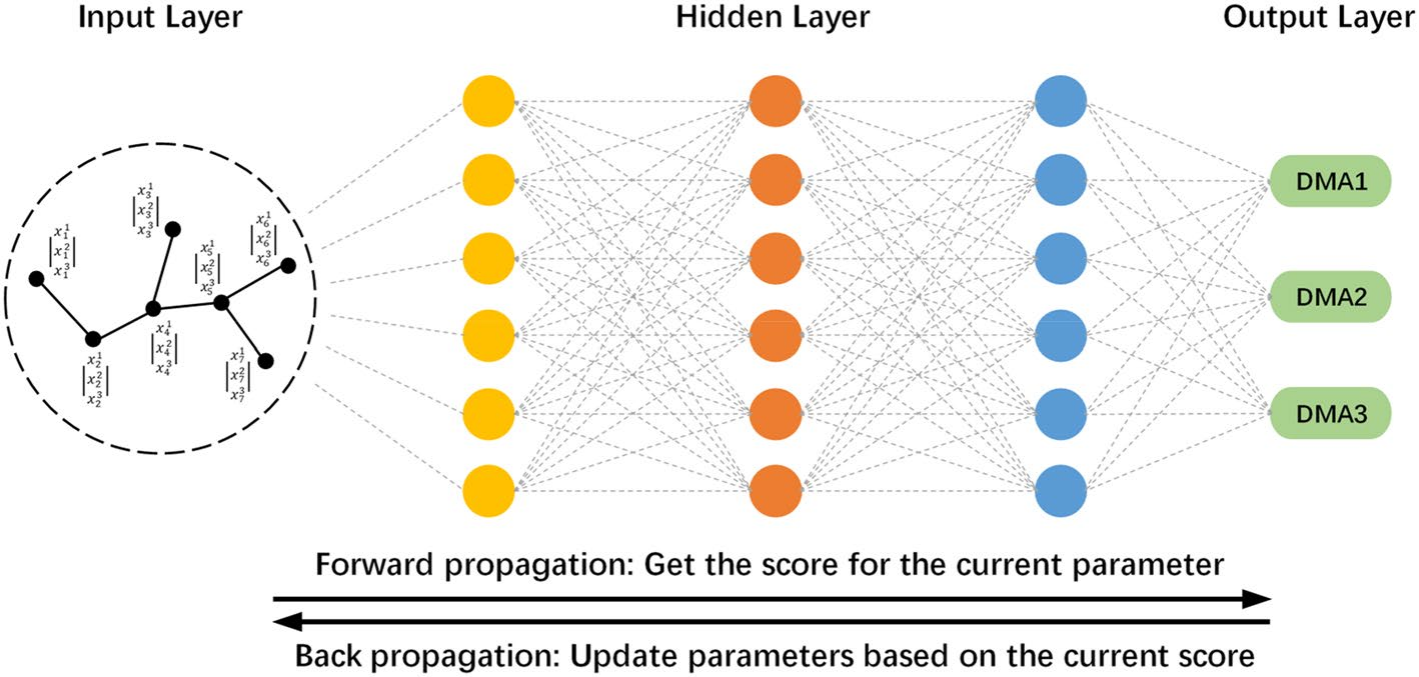
Schematic of integrated WNP using GNN.

The proposed structure of the GNN and the training process are shown in Fig. 4.

#### Build neural networks

The classification neural network of the WNP is composed of three parts: the input layer, hidden layer, and output layer. The input layer contains the information of the nodes, including information regarding the node characteristics and quantities. The hidden layer is composed of several layers, each of which is composed of several neurons, and the node parameters are iteratively optimized through training. The output layer is the WNP result under the ultimate network, and the evaluation index is used to evaluate the results of the classification. The neural network will use gradient descent to optimize the weights and bias of neurons, and then make the result of WNP as close as possible to the optimal score. After several iterations of optimization, the most reasonable result can be output.

#### Establish the evaluation index

The evaluation index ${I}_{e}$ is an indicator measuring the pros and cons of the WNP. It is used to evaluate whether the WNP results are suitable for subsequent practical application, and the evaluation index directly affects the results of the WNP through the neural network. WNP is evaluated based on the following indices:

- Resilience index $${I}_{r}$$
- Water quality index ${I}_{q}$
- Aggregation index $${I}_{a}$$
- Balance index $${I}_{b}$$

The resilience index ${I}_{r}$ was proposed by Todini to evaluate the resilience of a WDS under abnormal conditions^51^.

1 $$\begin{array}{c}{I}_{r}=\frac{\sum_{i=1}^{{n}_{n}}{Q}_{i}\left({h}_{i}-{h}^{*}\right)}{\sum_{r=1}^{{n}_{r}}{Q}_{r}{H}_{r}-\sum_{i=1}^{{n}_{n}}{Q}_{i}{h}^{*}}\end{array}$$

where ${n}_{n}$ and ${n}_{r}$ are the numbers of demand nodes and reservoirs, ${Q}_{i}$ and ${h}_{i}$ are the water demand and pressure head of node $i$, ${Q}_{r}$ and ${H}_{r}$ are the water discharge and total head of source point $r$, and ${h}^{*}$ is the design minimum pressure head of the network.

The water quality index is used to evaluate the age of the water in the WDN. Water age affects the content of chlorine in WDNs and thus affects the quality of water. Further research shows that the installation of valves at the boundary pipes to close water will increase the water age at the boundary, but the impact on the overall water quality is not significant^9,45^.

The aggregation index ${I}_{a}$ is used to evaluate the reasonableness of WNP. Similar spatial locations of demand nodes in the same DMA indicate that the total length of the internal pipe sections of the DMA is shorter and the difference between nodes in the DMAs is smaller.

2 $$\begin{array}{c}{I}_{a}=\sum_{j=1}^{{n}_{a}}\frac{{Q}_{j}\sqrt{{\left({x}_{j}-{x}^{*}\right)}^{2}{+\left({y}_{j}-{y}^{*}\right)}^{2}}}{\sum_{i=1}^{{n}_{n}}{Q}_{i}\sqrt{{\left({x}_{i}-{x}_{j}\right)}^{2}+{\left({y}_{i}-{x}_{j}\right)}^{2}}} \#\end{array}$$

where ${n}_{n}$ and ${n}_{a}$ are the numbers of demand nodes and DMAs, ${x}_{i}, {y}_{i}$, and ${Q}_{i}$ are the lateral position, vertical position, and water demand of node $i$, ${x}_{j}, {y}_{j}$, and ${Q}_{j}$ are the mean lateral position, mean vertical position, and total water demand of the $j$th DMA; and ${x}^{*}$ and ${y}^{*}$ are the mean lateral position and mean vertical position of all nodes. In the actual operation,$x$ and $y$ were aggregated results according to the connection relation of pipelines, which means that the two points connected by pipelines have closer $x$ and $y$ than before after Sect. 3.1, so as small as possible ${I}_{a}$ also ensures fewer boundary pipelines.

Another indicator used to evaluate the reasonableness of WNP is the balance index ${I}_{b}$. Similar pressure and water demand values between different DMAs indicate that the daily monitoring and maintenance costs of DMAs are lower.

3 $$\begin{array}{c}{I}_{b}=\sqrt{\frac{{n}_{a}-1}{\sum_{j=1}^{{n}_{a}}\left({Q}_{j}-\overline{Q }\right)}} \#\end{array}$$

where ${n}_{a}$ is the number of DMAs, ${Q}_{j}$ is the total water demand of the jth DMA, and $\overline{Q }$ is the mean water demand of all DMAs. The WDN evaluation index ${I}_{e}$ is established based on ${I}_{r}$, ${I}_{a}$, and ${I}_{b}$:

4 $$\begin{array}{c}{I}_{e}=\alpha {I}_{r}+\beta {I}_{a}+\gamma {I}_{b} \#\end{array}$$

where $\alpha$, $\beta$ and $\gamma$ are the weights of ${I}_{r}$, ${I}_{a}$ and ${I}_{b}$. This function is used to evaluate the training results of the GNN and indicate the update direction of the neural network parameters.

#### Train neural networks

In the neural network training stage, the model parameters are iteratively optimized until the expected result is obtained or ${I}_{e}$ converges. In each iteration of the optimisation process, the changes in the hidden layer node parameters lead to different WNP results and thus affect ${I}_{e}$. The partial derivative of ${I}_{e}$ is obtained by the compound function chain derivation rule, and the iteration direction and the step size of the parameter of the nodes at each hidden layer are calculated by back propagation.

Integrated WNP takes the ${I}_{e}$ values of the WNP targets and minimizes the negative impact of the WNP on the WDN. Compared with traditional two-step WNP, integrated WNP is more reasonable in terms of boundary pipes positions, and boundary closure has less impact on the WDN. Thus, it is more suitable for subsequent dynamic DMA boundary management.

### Dynamic DMA boundary management

In the traditional WNP, boundary pipes no longer change state once the division is complete. This causes an overall water pressure drop in the DMAs when a sudden situation occurs that consumes a large amount of water. Giudicianni et al. proposed the creation of a dynamic WNP method that allows a fixed DMA boundary to be opened in emergencies^48^; that is, multiple DMAs are combined into a large DMA, which overcomes the shortcomings caused by WNP.

When a node in the DMAs bursts with a large amount of water, the optimal strategy is to open some boundary pipes near the node. In contrast opening a pipe that is far away from the abnormal node costs more energy and may not obtain good results. This means that each boundary pipe needs to be analysed and classified into three categories: normally open, normally closed, and dynamic.

This paper proposes a dynamic method of managing the boundary pipes of DMAs. By calculating the influence factor $IF$ of the boundary pipes, the threshold values and boundary pipe states were set. Furthermore, dynamic pipes with higher impact factors are preferentially opened during emergencies.

5 $$\begin{array}{c}IF=\sum_{i=1}^{k}\frac{{Q}_{i}}{{L}_{i}}\#\end{array}$$

where $m$ is the statistical range (for any point, the $k$ vertices near it are considered in the $IF$ calculation), ${Q}_{i}$ is the water demand of node $i$, and ${L}_{i}$ is the shortest pipe length from the statistical point to node $i$.

## Experimental study

**Figure 5:**
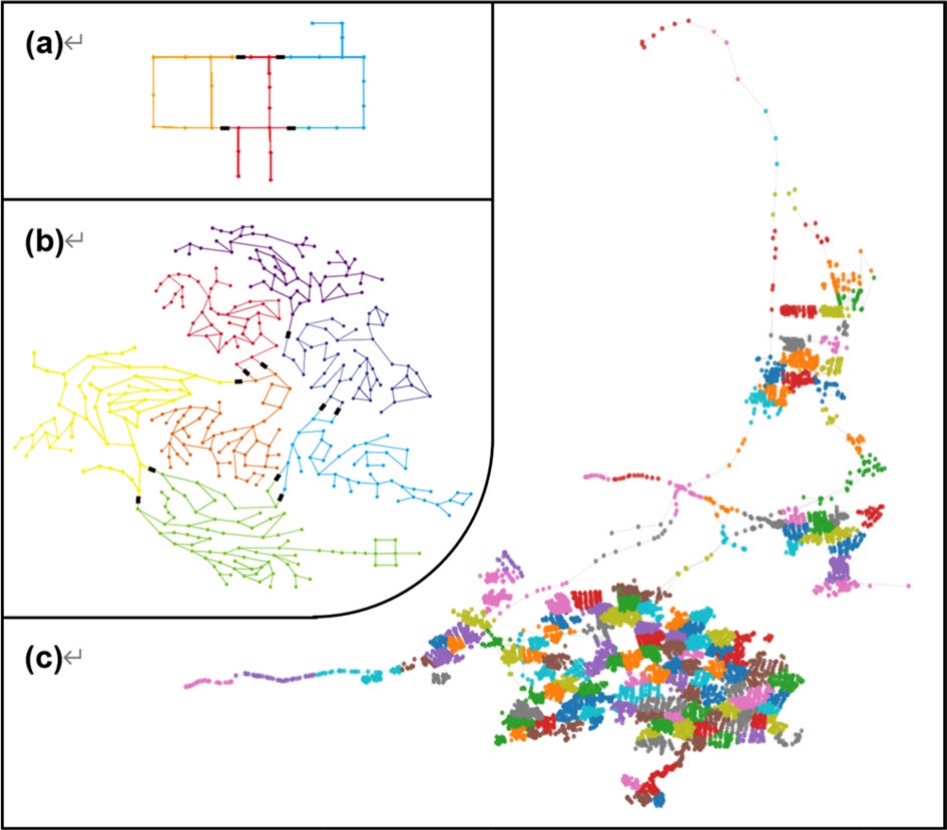
The water network structure and partitioning results (**a**) Hanoi 's water network (**b**) C-Town's water network (**c**) E-Town's water network.

This method was tested on a medium-sized WDN in Hanoi, C-town and E-town^65^. The Hanoi network is a part of the total Hanoi water distribution network, which includes 31 water demand nodes, and 50 pipes, as shown in Fig. 5a. The C-town network was used as a real water network in “The Battle of the Water Calibration Network”, containing 388 nodes, 429 pipes, one reservoir, and seven tanks, as shown in Fig. 5b. The E-town network is a large water network for supplying water to 400,000 people. It consists of five water sources, 11,063 water demand nodes, and 13,896 pipes, as shown in Fig. 5c. The model used the PyTorch framework to implement an improved form of WNP and simulated the hydraulic performance using EPANET 2.

### Case study on integrated WNP

To partition the water network of C-town to facilitate management and leakage control, the differences in the number of boundary pipes ${N}_{b}$ were compared, boundary pipes with flow meters ${N}_{f}$, boundary pipelines with valves ${N}_{v}$, and the minimum pressure head ${h}_{min}$, mean pressure head ${h}_{mean}$, and other indicators between the WDN before and after the partition. The simulation results of WNP are shown in Table 1 to illustrate its impact on the hydraulic performance of the WDN.

**Table 1:** Main characteristics and hydraulic performance after WNP.

	${N}_{b}$	${N}_{f}$	${N}_{v}$	${h}_{min}$ (m)	${h}_{mean}$ (m)	${I}_{r}$	${I}_{e}$
**Hanoi's water network**							
Unpartitioned	–	–	–	29.09	57.43	0.72	–
3 DMA (k = 3)	4	2	2	26.72	56.21	0.68	1.65
3 DMA (k = 5)	4	2	2	26.36	55.02	0.67	1.59
2 DMA (k = 3)	3	2	1	27.75	56.47	0.70	1.50
4 DMA (k = 3)	6	4	2	24.78	55.67	0.64	1.51
GT-WNP	5	3	2	25.40	56.19	0.67	1.07
CS-WNP	5	3	2	25.14	55.84	0.66	1.22
**C-Town's water network**							
Unpartitioned	–	–	–	22.07	57.69	0.73	–
7 DMA (k = 3)	10	6	4	21.78	56.58	0.69	1.64
7 DMA (k = 5)	10	6	4	21.74	56.04	0.68	1.62
5 DMA (k = 3)	7	4	3	21.79	56.93	0.71	1.48
9 DMA (k = 3)	16	8	8	19.51	54.95	0.66	1.54
GT-WNP	13	9	4	16.12	56.55	0.67	1.07
CS-WNP	14	9	5	20.93	56.56	0.68	1.45
**E-Town's water network**							
Unpartitioned	–	–	–	21.82	47.02	0.70	–
170 DMA (k = 3)	1451	863	588	21.81	45.74	0.65	1.61
170 DMA (k = 5)	1477	872	605	22.00	46.13	0.63	1.54
160 DMA (k = 3)	1369	835	534	21.40	46.05	0.68	1.46
180 DMA (k = 3)	1541	907	634	19.83	43.94	0.60	1.53
GT-WNP	1498	890	608	16.09	46.63	0.63	1.01
CS-WNP	1531	913	618	18.11	44.17	0.65	1.41

According to Fig. 5, WDNs were divided into 3, 7, and 170 DMAs respectively. $IF$ of each boundary pipe were calculated, and installed flow meters on the boundary pipes with $IF$ values greater than 0.12, and installed valves on the remaining boundary pipes. The simulation shows that all nodes meet the ${h}^{*}=20\mathrm{ m}$, and the partitioned Hanoi 's water network (${h}_{min}=26.72\mathrm{ m}$, ${h}_{mean}=56.21\mathrm{ m}$, ${I}_{r}=0.68$, and ${I}_{e}=1.65$), C-Town's water network (${h}_{min}=21.78\mathrm{ m}$, ${h}_{mean}=56.58\mathrm{ m}$, ${I}_{r}=0.69$, and ${I}_{e}=1.64$), and E-Town's water network (${h}_{min}=21.81\mathrm{ m}$, ${h}_{mean}=45.74\mathrm{ m}$, ${I}_{r}=0.65$, and ${I}_{e}=1.61$) met the daily needs. These values show that the water networks after partitioning with GNN-WNP had better resilience and evaluation indices than WNP based on graph theory (GT-WNP)^49^ and community structure method (CS-WNP)^32^, DMAs based on GNN-WNP had fewer boundary pipes, which means that using this method to set up DMAs requires less cost; compared with the ${I}_{r}$ of the water network without partitioning, that of this network is only 5.5% lower averagely.

**Figure 6:**
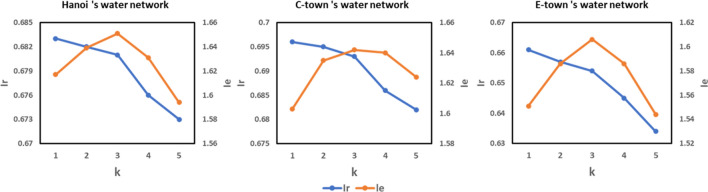
Influence of the aggregate node information process on WNP.

Next, the experiment focused on the influence of the aggregate node information process on WNP. The aggregate node information parameter $k=3$ was the most suitable for WNP (Fig. 6). This is because the 3-order attribute balanced the antagonistic relationship between the aggregation of attributes in each DMA and the separation of attributes between different DMAs.

**Figure 7:**
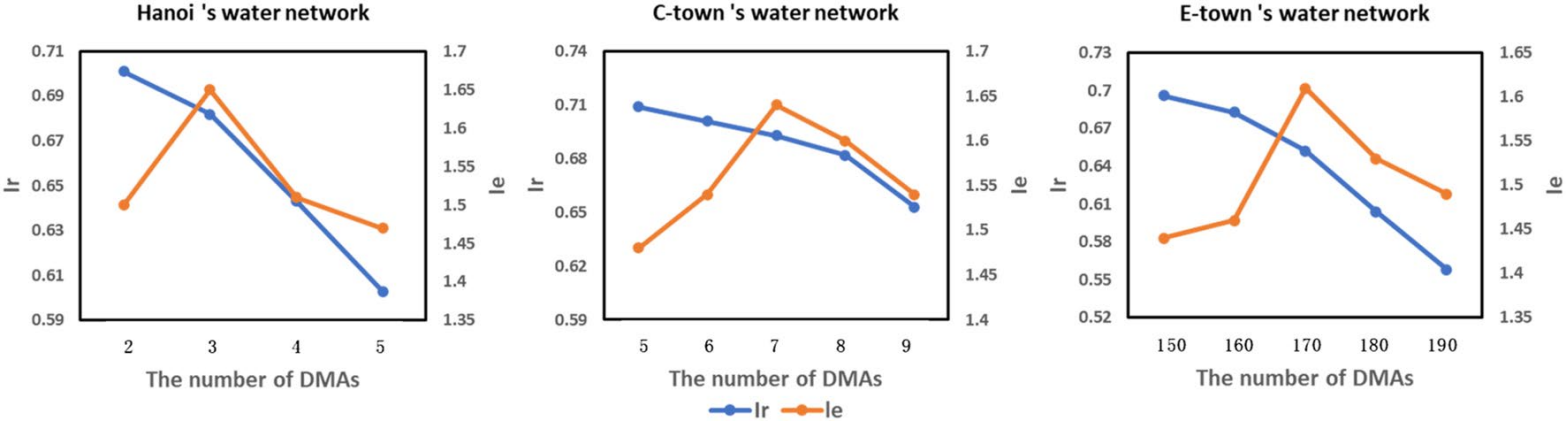
Influence of the number of DMAs on WNP.

In addition, the experiment focused on the influence of the number of DMAs on WNP. Using more DMAs is conducive to locating the leak location. However, in this case, more boundary pipelines were closed, which affected the hydraulic performance. Comparing the ${I}_{r}$ and ${I}_{e}$ values of different numbers of DMAs (Fig. 7), the hydraulic performance of the WDN dropped sharply when there were more than 3, 7, and 170 DMAs, meaning that using 3, 7, and 170 DMAs were suitable choices.

The main disadvantage of fixed DMAs is that the emergency capability of the WDN is reduced because of the closure of boundary pipes. When an emergency occurs (such as a large amount of water being used by fire-fighting equipment), regional water inflow restrictions lead to a decrease in overall water pressure and affect regional water use. Therefore, this study explores the possibility of using dynamic DMA boundary management to overcome the disadvantages of fixed WNP.

### Case study on integrated WNP and dynamic DMAs

**Figure 8:**
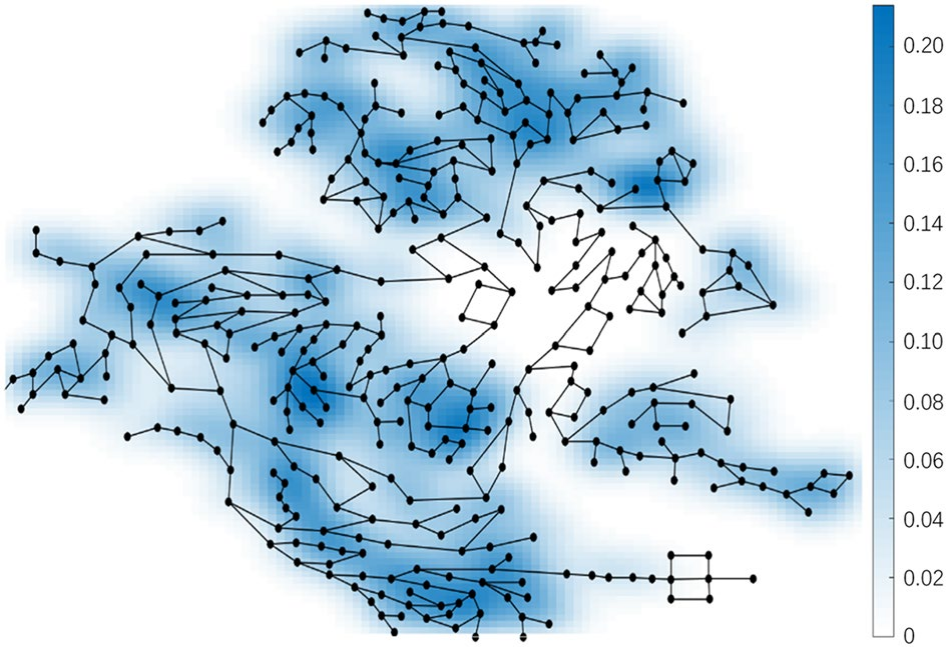
Weights of C-Town's pipes.

The dynamic DMAs evaluate the daily status and emergency response status of the boundary pipes by calculating $IF$, and the $IF$ of the boundary pipes of C-town are shown in Fig. 8. $IF$ mainly refers to the impact of the pipes on the capacity of the WDS to recover from abnormal situations during the opening or closing conversion process. Different measures were taken for different pipes, divided into the following three categories:

- Install flowmeters in boundary pipes with high $IF$ values.
- Install fixed valves in boundary pipes with low $IF$ values.
- Install dynamic valves in boundary pipes with medium $IF$ values.

The WDN of C-town is simulated to determine the boundaries of DMAs and the opening or closing schemes of dynamic boundary pipes under different conditions. The $IF$ values of the DMA boundary pipes changed most rapidly between 0.07 and 0.12. Therefore, flow meters were installed in the boundary pipes with $IF$ values higher than 0.12, fixed valves were installed in the boundary pipes with $IF$ values lower than 0.07, and dynamic valves were installed in the boundary pipes with $IF$ values between 0.07 and 0.12.

The dynamic DMA configuration scheme for three emergencies (small, medium, and large fires) simulated in the WDS of C-town is shown in Table 2. Comparing the dynamic DMAs and the fixed DMAs, both networks maintained effective operation in the case of the small fire without opening the boundary dynamic pipes; ${h}_{mean}$ = 54.21 m and ${I}_{r}$ were reduced by 6.9% compared to the unpartitioned layout.

**Table 2:** Main characteristics and hydraulic performance of fixed DMAs and dynamic DMAs in different emergency situations.

	${N}_{f}$	${N}_{v}$	${h}_{mean}$	${h}_{min}$	${I}_{r}$
Unpartitioned	–	–	57.69	22.07	0.73
**Small fire**					
Unpartitioned	–	–	54.33	21.29	0.68
Fixed	6	4	54.21	21.29	0.68
Dynamic	6	4	54.21	21.29	0.68
**Medium fire**					
Unpartitioned	–	–	53.54	17.56	0.67
Fixed	6	4	51.72	15.98	0.65
Dynamic	7	3	52.37	15.54	0.67
**Large fire**					
Unpartitioned	–	–	52.29	14.37	0.66
Fixed	6	4	42.56	9.52	0.54
Dynamic	7	3	50.15	13.23	0.63

In the case of the medium fire, the dynamic WNP maintained normal operation and the fixed WNP fell into an abnormal situation. Compared with the unpartitioned water network, the ${I}_{r}$ values of the dynamic and fixed DMAs decreased by 9.6% and 12.3%, respectively, and the ${h}_{mean}$ values of the dynamic and fixed WNP were 52.37 m and 51.72 m, respectively.

In the case of the large fire, the resilience of the dynamic DMAs was much stronger than that of the fixed DMAs. Compared with the unpartitioned water network, the ${I}_{r}$ values of the dynamic DMAs and the fixed DMAs decreased by 13.7% and 26%, respectively, and the ${h}_{mean}$ values of the dynamic and fixed WNP were 50.15 m and 42.56 m.

Thus, the dynamic DMAs were shown to have significantly stronger resilience under emergency conditions than fixed DMAs.

### Ethical approval

The manuscript is conducted within the ethical manner advised by the journal.

## Conclusion

This paper presents a new method which can realize integrated WNP using dynamic DMAs. This method is based on graph neural network technology, which is often used for the classification of graph data; influence evaluation technology, which is often used for data importance ranking; and hydraulic simulation, which is often used for the rationality assessment of water network partitioning. A framework of integrated WNP and dynamic DMAs was developed based on PyTorch to apply the described method to a simulated medium-sized water distribution network called C-town, and used general indicators (such as ${I}_{r}$) to verify the rationality of the partition. Through the simulation, the impact of different model parameters on WNP was compared based the hydraulic performance of dynamic DMAs with that of fixed DMAs. The simulation results show that partitioning the water network using a graph neural network can provide an excellent, interpretable, and fast solution. Furthermore, this method provides a reliable basis for dynamic DMA boundary management and proves that dynamic DMAs have far better hydraulic performance than fixed DMAs in emergencies.

Future work will focus on solving the problem of DMA interactivity. Specifically, when DMAs in the water network are connected in a loop. Therefore, a single boundary pipe does not only affect the two DMAs connected to it. Evaluating the hydraulic impact of the boundary pipes on the overall network will help to determine the optimal plan under emergency conditions and improve the efficiency of boundary dynamic operations.

## Data Availability

Data can be shared upon request from the corresponding author.
